# Energy and Distance-Aware Hopping Sensor Relocation for Wireless Sensor Networks

**DOI:** 10.3390/s19071567

**Published:** 2019-04-01

**Authors:** Moonseong Kim, Sooyeon Park, Woochan Lee

**Affiliations:** 1 Department of Liberal Arts, Seoul Theological University, Bucheon 14754, Korea; moonseong@stu.ac.kr; 2 Department of Electrical Engineering, Incheon National University, Incheon 22012, Korea; anisoo@inu.ac.kr

**Keywords:** hopping sensor, jumping robot, mobile sensor, relocation protocol, energy and distance-aware relocation protocol, Internet of Things (IoTs), Wireless Sensor Networks (WSNs)

## Abstract

Recent advances in big data technology collecting and analyzing large amounts of valuable data have attracted a lot of attention. When the information in non-reachable areas is required, IoT wireless sensor network technologies have to be applied. Sensors fundamentally have energy limitations, and it is almost impossible to replace energy-depleted sensors that have been deployed in an inaccessible region. Therefore, moving healthy sensors into the sensing hole will recover the faulty sensor area. In rough surfaces, hopping sensors would be more appropriate than wheel-driven mobile sensors. Sensor relocation algorithms to recover sensing holes have been researched variously in the past. However, the majority of studies to date have been inadequate in reality, since they are nothing but theoretical studies which assume that all the topology in the network is known and then computes the shortest path based on the nonrealistic backing up knowledge—The topology information. In this paper, we first propose a distributed hopping sensor relocation protocol. The possibility of movement of the hopping sensor is also considered to recover sensing holes and is not limited to applying the shortest path strategy. Finally, a performance analysis using OMNeT++ has demonstrated the solidification of the excellence of the proposed protocol.

## 1. Introduction

Big data includes comprehensive technologies which analyze and add values to a large amount of data. Novel techniques for collecting and transmitting data have become very important along with the advance of big data technology [1]. Therefore, networking between sensor nodes plays a great role in progressing big data industry [2,3]. Several decades have witnessed the dramatic rise of wireless sensor network technology to exploit environment, weather, and battle field information.

A wireless sensor network has a wide variety of applications under radioactive contaminated regions, battle fields, and/or a region of disaster [4,5]. It is very hard to approach these regions due to harmful chemicals or unavoidable obstacles. Moreover, hundreds of sensors deployed in an enemy site gathers a lot of valuable military information. The scattering of the sensors through wide and rough surfaces is of critical importance to collect valuable data especially in unmanned area.

However, the small-sized sensors have a limitation of energy due to the small volume of battery capacity. Some sensors consume their energy quicker than other nodes due to an uneven access to those sensors. In the worst case, the communication between sensors might shut down due to an energy-depletion of only a few nodes. To avoid the unexpected shutting down of the whole network, several energy-efficient schemes have been proposed. Rerouting or multipath by load-balancing are some noticeable schemes in this direction of research [6,7,8].

A region which is not covered by a data-collecting sensor node is called a “sensing hole”. That is, as the number of faulty sensors increases, sensing holes emerge [9]. The main reason of this sensor death is power depletion. Several schemes have been proposed to prevent the appearance of sensing holes. These schemes include sensor state (active/idle) control and efficient path routing. Mobile sensors are able to approach dangerous area autonomously and replace dead sensor nodes. Initial studies for the mobile sensors were just focusing on the sensor relocation capability. In this scheme, fixed sensors scattered in a Wireless Sensor Networks (WSN) region are responsible for guiding mobile sensors to the wanted directions. Reference [10] demonstrates a mobile network structure in which wheeled mobile devices replace malfunctioned fixed nodes. However, the wheel-based movement is not suitable for rugged surfaces which prevent fast deployment, hence it leads to a quick power consumption.

**Figure 1:**
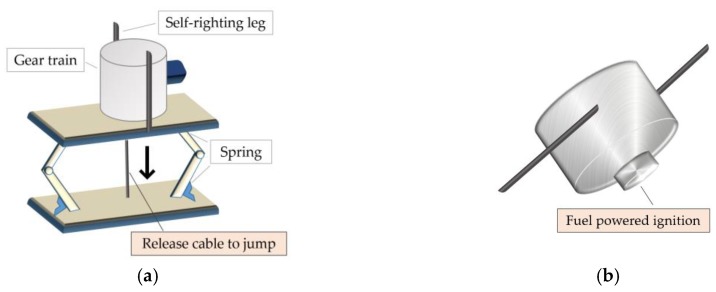
Examples for robots based on hopping: (**a**) MSU [11]; (**b**) DARPA [12,13].

Hopping-based relocation is proposed to overcome the limitation of the wheel-based scheme. A hopping sensor node is designed to jump to a specific direction which can be steered by the users. Reference [11] demonstrates some specifications of the jumping robots, as shown in Figure 1a, including the maximum height and operational range. For another example, the mobile unit, as shown in Figure 1b, funded by DARPA is able to reach a maximum 10-m height and hop 100 times with one fuel injection [12,13].

Sensor relocation to recover sensing holes by mobile sensors has attracted many researchers. The authors of Reference [14] propose a sensor relocation scheme based on the shortest path manner. However, specific sensors on the shortest path are frequently accessed, and thus additional sensing holes can be generated by these sensors. To remedy this problem, Reference [15] proposes a multipath scheme instead of the single shortest path policy. Reference [15] lowers the uneven movement of hopping sensors which causes a fast energy exhaustion of specific sensors. Reference [15] also improves the ratio of operational hopping sensors in the network as well as the success ratio of sensor relocation, compared to the shorted path method.

The sensing holes arise randomly, and the appearance of them is not limited to the specific region. It is also possible that multiple holes arise simultaneously. A repetition of the shortest path relocation can be used to cover multiple sensing holes. However, the possibility of sensing hole appearance becomes higher when the specific path is repeatedly accessed. Reference [16] suggests a disjointed relocation path when the sensing hole is generated. Thus, it enhances the mobility-capable sensor ratio through the network coverage. Reference [17] takes into consideration the category of sensing field like rock or mud. The shortest path scheme is not taken seriously in this manner.

Past researches include the effectiveness of the shortest path method, the multipath method, and the quality of the path method to recover or conceal the sensing hole. However, in these past researches, every cluster header is forced to acknowledge all the network information to route the path for the recovery. Actually, the overhead to maintain all the information of the network is quite burdensome and induces a lot of control message transaction.

Our proposed protocol does not need to obtain the network managing information from the adjacent cluster header and/or whole network for the current cluster header to recover a sensing hole. The current cluster header is enough to recover the sensing hole by requesting sensor nodes to adjacent cluster header. Cluster headers may direct sensor nodes in their cluster range to the neighbor sensing hole. When the cluster header selects sensor nodes to be moved, the remaining energy of the sensor nodes and the distance from the sensing hole are considered. In previous researches, almost all results were obtained using the relocation path through an unrealistic sensor network topology which is hard to be known. However, it is very important that this study is the first simulation using OMNeT++ which reflects actual environments, as far as we know.

The structure of our paper is as follows. Section 2 demonstrates the detailed operation of proposed relocation protocol. The simulation results and performance comparisons are described in Section 3. Section 4 concludes this paper.

## 2. The Proposed Protocol to Relocate Hopping Sensors

The detailed operation of the proposed hopping sensor relocation protocol is demonstrated in this section. First, we assume the basic specification required in the hopping sensor network. We also defined the message types for the proposed protocol and detailed the examples of a sensing hole recovery using the message sequence diagram.

### 2.1. Characteristics and Assumptions of Hopping Sensors

The feature of the hopping sensor is that it can move in a rough surface region such as rock or sand by jumping rather than using a wheel. In addition, the data transmission radius of the hopping sensor node can be expanded by jumping. The authors of References [18,19] explained that the sensor node can jump to the appropriate height and adjust the communication radius. Reference [18] introduced the “Airborne communication scheme” in which the communication radius was expanded by jumping when two hopping sensors were out of the communication radius on the ground. In Reference [19], when the sensor node jumped 1 m from the ground, they showed a six-fold increase of the radius compared with that on the ground. The connectivity of the sensor nodes to the entire network could be improved in this way, and a method for covering the whole area of the network was proposed. The authors of Reference [20] actually implemented the hopping sensor, using a launcher for the jump, and measured the transmission radius. In Reference [20], the variation of the transmission radius according to the height was compared with the result of Reference [19] and confirmed that the two results were similar.

In the research area of wireless sensor network, there are various algorithms for clustering the network area and selecting the headers [21]. It is assumed that the clustering and the selection of the headers have already been set up, since the objective of this paper is to research a method to efficiently relocate mobile hopping sensor nodes.

**Figure 2:**
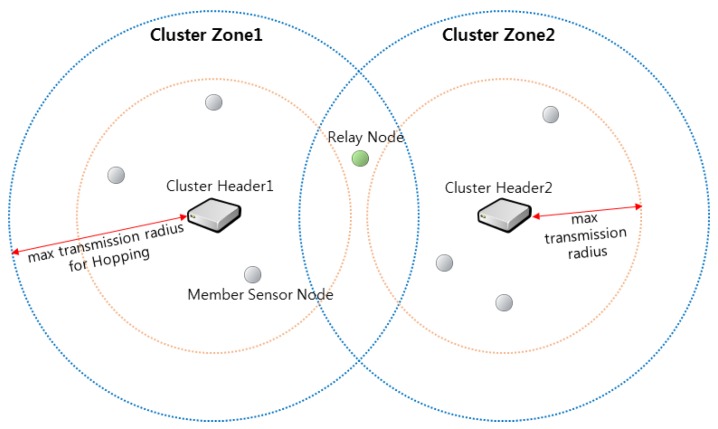
The definition of the terms in hopping sensor networks.

In detail, the sensor nodes are randomly distributed in the area where the initial data is desired to be collected. The network area is then divided into clusters by various algorithms, and a cluster header suitable for each cluster is selected. The cluster header communicates periodically with the sensor node members in its cluster. The cluster headers can jump to extend the transmission radius to establish a connection with the relay node in Figure 2. Furthermore, sensor nodes include a GPS unit that can identify their current position [22].

Figure 2 briefly describes each term used in this paper. The sensors in this figure are different in shape and color, but in fact, they are all the same sensors, and all sensor nodes are hopping sensor nodes. Since the sensor nodes can move while jumping, the sensors are called as “hopping sensors”. The hopping sensor may be a “cluster header” or a “cluster member node”. Since the maximum transmission radius when the cluster headers take the highest jump is defined as a “cluster zone”, communication between the cluster headers cannot be directly established. Some member sensor nodes in the overlapping region of the neighbor cluster zone can communicate with two or more cluster headers. Their role is similar to those of helper nodes in the middle of a routing path between two nodes to increase the reliability of communication [23]. In this paper, we will refer to this sensor node as a “relay node”, and the role of the relay node will play a role in intermediating the communication between the cluster headers. Again, a hopping sensor jumps only when moving and when communication between a cluster header and a relay node is made.

### 2.2. Components and Brief Descriptions of the Proposed Protocol

**Figure 3:**
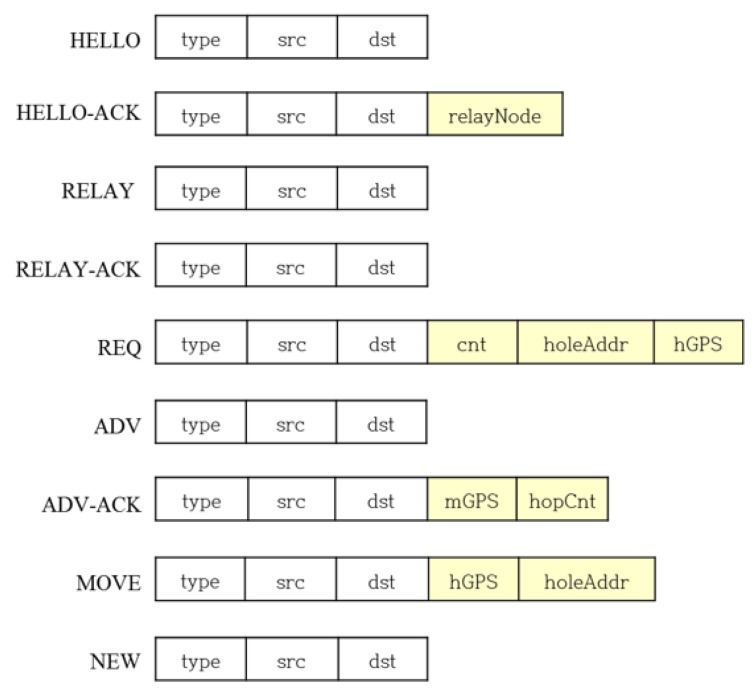
The message types for the proposed protocol.

It is assumed that the selection of the appropriate cluster headers has already been made among the sensor nodes deployed in the rugged and wide area where the data is desired to be collected. Each cluster header can jump to expand transmission range, and the entire region is clustered based on the maximum transmission radius as shown in Figure 2. In Reference [24], research has been conducted on how the transmission radius increases with the jumping height. The cluster header identifies the member sensor nodes in its cluster zone and uses the message types shown in Figure 3 to relocate the hopping sensor nodes.

- **HELLO message**: The cluster header periodically (using “helloMsgTimer”) broadcasts a HELLO message to identify its member sensor nodes. The member sensor node receiving HELLO checks the address of the HELLO transmitted (src) and registers it as its own cluster header. Also, if there are more than two registered addresses, the member sensor node becomes a relay node.

· type: HELLO
· src: The address of the header node that sends HELLO
· dst: Broadcast address

- **HELLO-ACK message**: In response to HELLO, the member sensor node receiving the HELLO message transmits HELLO-ACK to its cluster header.

· type: HELLO-ACK
· src: The address of the member sensor node transmitting the HELLO-ACK
· dst: src of HELLO message received
· relayNode: Set to “true” if the sensor node transmitting the HELLO-ACK is a relay node; otherwise, set to “false”.

- **helloMsgTimer**: The cluster header executes the timer helloMsgTimer for a predetermined time by itself after the HELLO transmission. When the timer expires, the cluster header computes the number of its member nodes using HELLO-ACKs, and if the member nodes number is less than a certain number, it can determine that its zone could be a sensing hole. It periodically broadcasts HELLO for sensing hole identification and executes the timer helloMsgTimer again.
- **RELAY message**: If the member sensor node of the header is insufficient (i.e., a sensing hole), the header broadcasts a RELAY message to all its relay nodes.

· type: RELAY
· src: The address of the header that sends the RELAY message
· dst: Broadcast address

- **RELAY-ACK message**: The relay node sends a RELAY-ACK response message to the cluster header that sent the RELAY.

· type: RELAY-ACK
· src: The address of relay node sending RELAY-ACK
· dst: src of RELAY message received

- **REQ (Request) message**: The REQ is a message that the cluster header, in which the sensing hole occurs, requests a relocation of the member sensor nodes to neighboring cluster zones. The cluster header of the sensing hole transmits a REQ message to the relay node using the first received RELAY-ACK. The relay node that receives the REQ transmits the REQ to any one of the cluster headers that is not the sending address (src) of the received REQ among the cluster headers of the relay node. After a cluster header of the sensing hole transmits a REQ, if the cluster header receives a RELAY-ACK from another relay node, the newly received RELAY-ACK is ignored.

· type: REQ
· src: The address of the header of the sensing hole that transmits the REQ or the address of the relay node when forwarding REQ
· dst: src of the RELAY-ACK received or the address of the cluster header of the relay node when it forwards REQ message
· cnt: Number of member sensor nodes required in the sensing hole
· holeAddr: The address of the header where the sensing hole occurred
· hGPS: Location information of the sensing hole header

- **ADV message**: A cluster header neighboring a sensing hole and receiving the REQ broadcasts an ADV to its member sensor nodes.

· type: ADV
· src: The address of the cluster header that sends the ADV
· dst: Broadcast

- **ADV-ACK message**: The member sensor node that received the ADV sends its ADV-ACK including its position and the currently available number of hops to its cluster header. The relay node does not transmit ADV-ACK.

· type: ADV-ACK
· src: The address of the member sensor node that transmits the ADV-ACK
· dst: src of ACK message received
· mGPS: Location information of member sensor node
· hopCnt: The number of currently available hops (i.e., the residual energy which can be used for moving)

- **MOVE message**: The cluster header that receives the ADV-ACKs selects the movable nodes by referring to 1) currently available hops (residual energy) and 2) the geographical distance between the member sensor node and the cluster header where the sensing hole occurs. The header sends MOVE to the selected member sensor nodes. The member sensor node receiving the MOVE is relocated to the cluster zone where the sensing hole occurs.

· type: MOVE
· src: The address of the header that sends the MOVE
· dst: The address of the member sensor node selected for relocating
· hGPS: Location information of the sensing hole
· holeAddr: The address of the header where the sensing hole occurred

- **NEW message**: The member sensor node that moved to neighboring cluster zone sends the NEW message to a new cluster header. Upon receiving the HELLO message after a predetermined time (helloMsgTimer), the relocated member sensor node can determine whether it is a relay node by initializing its header nodes.

· type: NEW
· src: The address of the member sensor node sending the NEW message
· dst: The holeAddr of the MOVE message received

The cluster header periodically communicates with its members in its cluster to determine the status of member sensor nodes. For example, if the number of answers to the HELLO message is less than a certain level, it is possible for the cluster header to recognize that its cluster zone could be a sensing hole. Also, the member sensor nodes keep their headers’ information by grasping the header IDs. If a sensor node has more than two header IDs, the node is a relay node. A relay node could maintain the maximum transmission distance by jumping.

**Figure 4:**
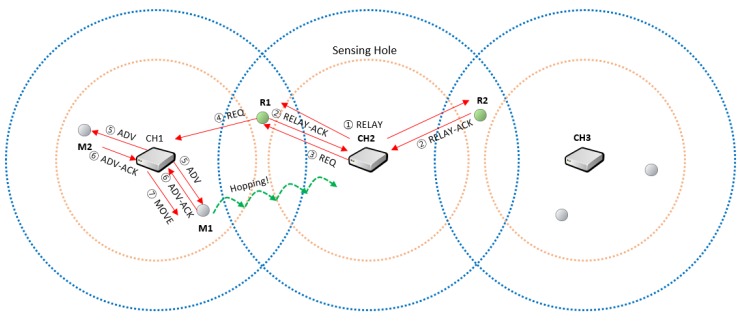
An example of hopping sensor relocation through the proposed protocol.

In Figure 4, we assume that the cluster header CH2 has determined that its zone is a sensing hole as the number of member sensor nodes becomes below certain number.

1. The cluster header CH2 sends RELAY message to the relay nodes (R1, R2) to select the relay node to forward the message.
2. Each relay node sends a reply message RELAY-ACK to the cluster header CH2 to indicate that it is possible to forward the request to the other cluster headers (CH1, CH3). In Figure 2, it is assumed that the RELAY-ACK of the node R1 first arrived at CH2. Therefore, another RELAY-ACK from the R2 node is ignored.
3. The cluster header CH2 receives the response message RELAY-ACK from R1 and then requests at the node R1 that one sensor node is needed. At this time, the location information of the cluster header CH2 is included in the REQ message.
4. Relay node R1 forwards the REQ, including the required number of hopping sensor nodes requested by CH2, to another cluster header CH1.
5. The cluster header CH1 sends an ADV message to select the nodes to move from its zone to the neighbor cluster zone.
6. Each member sensor node sends its location information and energy information, which are able to be used for moving, to its cluster header CH1.
7. The header CH1 receives the response message from each member sensor node and then selects the movable member considering the position information of the cluster header CH2 and the information (location and energy) of the respective members. At this time, the energy of each member and the distance between a member and the neighboring cluster zone can be considered concurrently. Specifically, the cluster header obtains the value of the following function (1) *f* (*node_i, w*) for each member sensor node. The member having the maximum value will be selected as the node to be moved.

*f*(node_*i*, *w*) = *w* *(*c_i/c_ini*) + (1 − *w*)*((*d_max* − *d_i*)/*d_max*) (1)

where *d_i* is the distance between the member sensor node *node_i* and the cluster header (CH2) of the zone to be moved, *c_i* is the number of movable hops (residual energy) of the node *node_i*, *c_ini* is the initial value for the number of movable hops, and *d_max* is *max* {*d_i*}. If *w* is close to 0, the distance to the moving zone has more weights in the selection of the candidate hopping sensors to move. On the other hand, if *w* is close to 1, the residual energy has more weights.

In Figure 4, the member sensor node M1 is selected considering the node position only, and the cluster header CH1 commands the member node M1 to move to the neighboring cluster zone.

**Figure 5:**
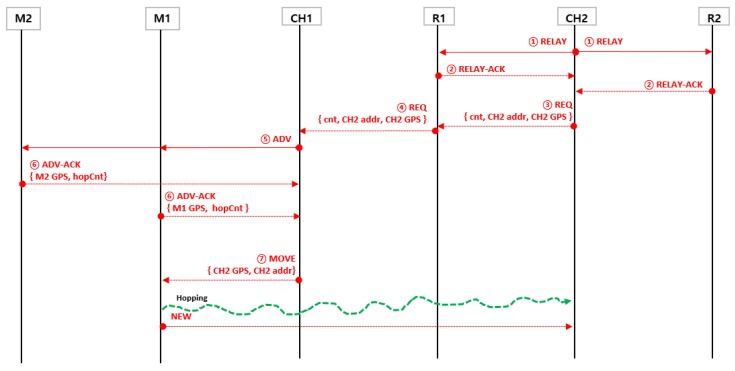
A message sequence diagram for the example in Figure 4.

8. The member sensor node M1 moves to the neighbor cluster and recovers the sensing hole. There is no need to communicate between sensor nodes while the member sensor node is moving. It only receives the relocation request from the current cluster header once and moves to the given coordinates without communication. After moving, communication with the new cluster header is resumed. Figure 5 illustrates the message flow for the example in Figure 4.

**Figure 6:**
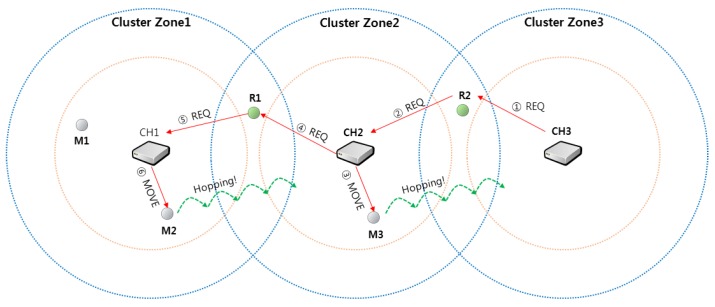
An example of recovering sensing holes through relay relocation strategy.

In order to recover cluster zone 3, which is a sensing hole as shown in Figure 6, the relay relocation strategy between clusters is shown as follows.

The cluster header CH3 transmits a message requesting a single sensor to the relay node R2.

1. Relay node R2 forwards the received message to the cluster header CH2 of cluster zone 2.
2. Cluster header CH2 selects M3 as a movable sensor node in its own zone and sends a move command to cluster zone 3.
3. The cluster header CH2 predicts that its zone may also be a sensing hole and sends a message requesting one sensor to relay node R1, sequentially.
4. The relay node R1 delivers the message REQ to the cluster header CH1 like ②.
5. Cluster header CH1 selects M2 among the sensor node members in its zone and commands M2 to move.

As a result, one sensor is assigned appropriately within each cluster zone so that all cluster zones can be recovered from sensing holes, sequentially. In addition, the sensor nodes M2 and M3 that have moved to the new zone can communicate with the cluster headers of each zone and receive a new ID. This procedure is out of the scope of this paper, so the details are omitted and Reference [25] can be referred to.

### 2.3. Basic Operation of Each Node-Specific Protocol for Sensing Hole Recovery

**Figure 7:**
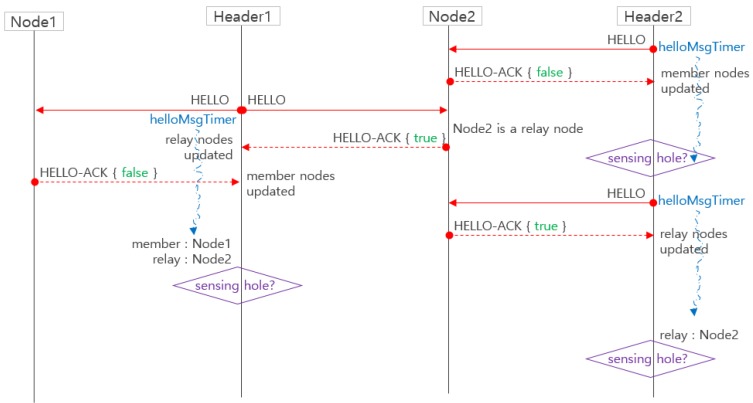
The managing members and relay nodes in cluster headers.

In Figure 7, all headers periodically broadcast a HELLO message to maintain the information of their member sensor nodes. A timer helloMsgTimer of a predetermined time is executed for periodic broadcasting. The member node adds the node that transmitted the HELLO as its own header and judges that it is a relay node when it has two or more own headers. In order to inform its header that the member node is a relay node, the member node sets the relayNode option to “true” in HELLO-ACK. The header that receives the HELLO-ACK adds the node that transmitted the HELLO-ACK as its own member and sets it as its own relay node if it is a relay node. If the number of members of the zone is less than a certain number, it can be determined that the cluster is a sensing hole.

**Figure 8:**
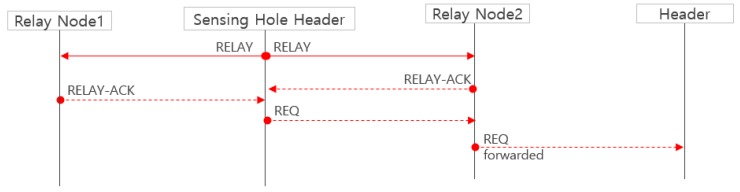
An attempt at recovering a sensing hole.

In Figure 8, if the header determines that its cluster is a sensing hole, it broadcasts a RELAY message to all relay nodes. The relay node receiving this message responds with a RELAY-ACK. The header sends an REQ message to the relay node that transmitted the first received RELAY-ACK. Successively, the relay node receiving the REQ delivers the REQ to its own header which is not in the sensing hole. However, it may happen that the cluster header of the sensing hole broadcasts the RELAY message but that a hopping sensor cannot be supplied for the sensing hole recovery.

First, a relay node transmits a REQ message to a neighbor cluster header, but neighboring zones may also be sensing holes. In this case, the cluster header of the first sensing hole repeatedly broadcasts the RELAY message after a predetermined time (helloMsgTimer) and fails to receive the NEW message of the hopping sensor newly entered. Thus, the cluster header of the first sensing hole could suspend broadcasting of the RELAY message for a certain period of time, hoping that the neighboring zone is recovered from the sensing hole.

Second, if all the relay nodes are dead, they will not receive the RELAY-ACK. In this case, the RELAY message is transmitted again after a predetermined time (helloMsgTimer). After a certain number of iterations, as in the first case, the broadcasting of the RELAY message could be stopped for a certain period of time. After the hopping sensors move from the neighboring zones, a sensor among these sensors is likely to become a new relay node, and a new protocol can be started from the new relay node.

**Figure 9:**
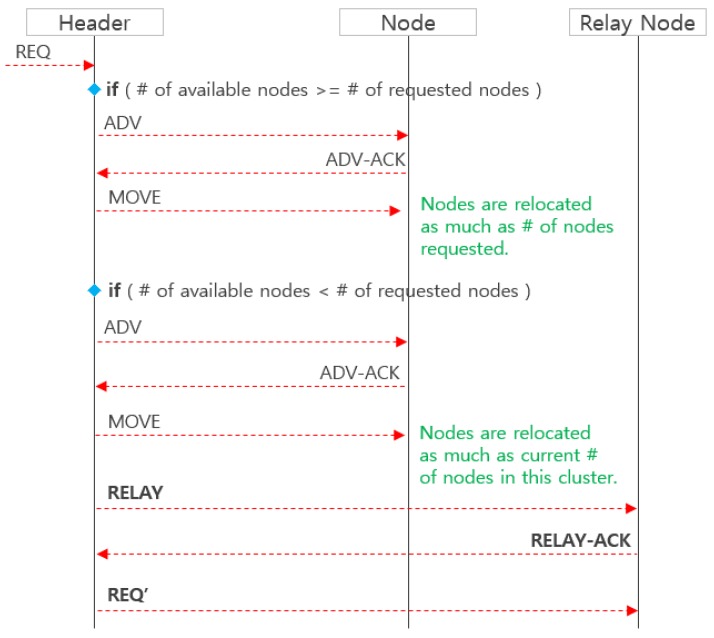
The relocation strategy of a cluster header receiving the REQ message forwarded from Figure 8.

When a header receives a REQ message from a relay node, it immediately checks the number of movable members in its own cluster and compares with the number of requested members set in the REQ message, as shown in Figure 9.

First, when the cluster has sufficient movable members more than the requested members, the ADV message is broadcasted to all its member nodes, and the member nodes receiving the ADV transmit the message ADV-ACK, including its location and energy information, to the header. The header receiving the ADV-ACK selects the movable members appropriately (with the weight was mentioned above in Section 2.2) and sends MOVE messages to the candidate members.

Second, when the number of movable members is insufficient, the movable member can be moved to the sensing hole as much as possible. Due to this moving, its zone may become a sensing hole. In order to avoid this situation, it is possible to repeat the process of Figure 8 by transmitting the RELAY and REQ’ to another relay node.

**Figure 10:**
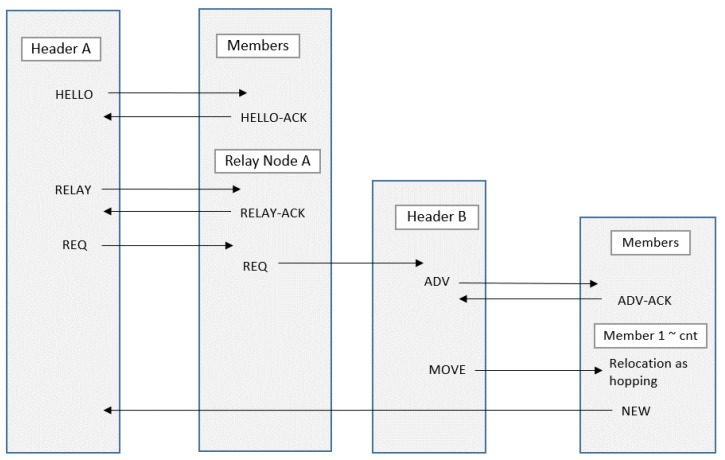
The message flow of the proposed hopping sensor relocation protocol.

Overall, the message flow of the hopping sensor relocation protocol described above is briefly summarized in Figure 10.

## 3. Simulation Results and Analysis

**Figure 11:**
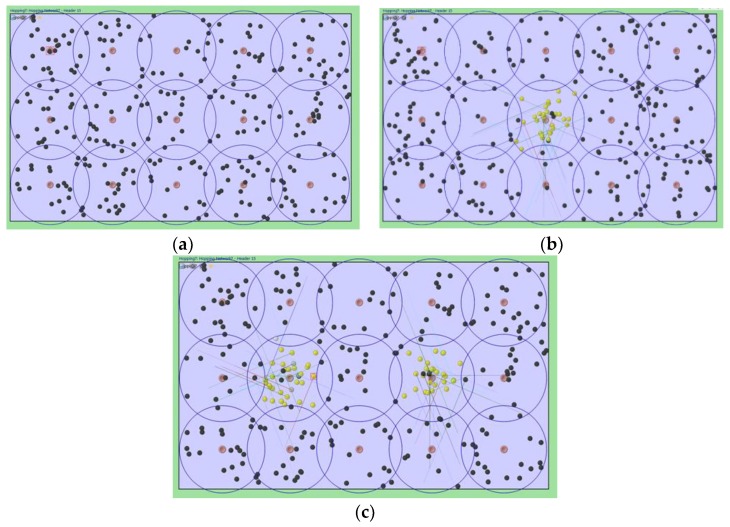
The wireless sensor network environment and examples for when sensing holes occur: (**a**) The hopping sensors are scattered, the cluster zones are configured, and the cluster headers are set up; (**b**) a single sensing hole occurred; and (**c**) two sensing holes occurred.

We have implemented the proposed relocation protocol for hopping sensors using the well-known network simulator OMNeT++ [26,27] to evaluate its performance. Table 1 describes the environment settings used in the simulation. As shown in Figure 11a, 285 hopping sensors were randomly distributed in the area of 250 m × 150 m to collect the data. As mentioned previously in Section 2.1, we assumed that 15 cluster zones and headers were set appropriately. If there are less than 5 sensor nodes in each cluster zone, it is assumed to be a sensing hole. The hopping sensor employs the IEEE 802.11 model, and the maximum communication radius by jumping is 29 m. Also, it is assumed that the sensors move 1 m by one hopping and are capable of hopping up to 290 times. Note that this is the distance that the hopping sensor can traverse diagonally across a given area.

The number of sensing holes is up to 2 as shown in Figure 11b,c. For example, Figure 11b shows a scenario that one sensing hole could occur, since the sensor nodes’ energies are abruptly consumed due to continuous data collection in the middle cluster. For this scenario, each sensor in the middle cluster performs data collection with an exponential distribution (average 10 simulation time units), and energy consumption follows a uniform distribution. When less than 5 sensors collecting data in the zone, the cluster header can recognize that a sensing hole is generated, by the periodic HELLO message. In order to simulate the relocation of member sensor nodes, the sensors in the other zones are assumed that there was no data collection. Figure 11 also shows that the yellow color sensor is in a fault state due to an energy depletion after continuous data collection. The movement of the hopping sensor is indicated by a solid line.

Most of the hopping sensor relocation protocols studied so far have considered only the shortest path between cluster zones, which is, in reality, very hard to implement in a wireless sensor network. In order to overcome this problem, we have implemented the simulation in a distributed environment. As far as we know, this is definitely the first simulation result in this research area using OMNeT++, which takes into account the distributed environment and all communication system layers similar to the real world.

The proposed relocation protocol considers both the distance to the sensing hole and the movability of the nodes. When the weight *w* is close to 0, only the distance to the sensing hole is considered, and when the weight *w* is close to 1, the residual energy is considered. Previous studies have considered the shortest distance between clusters, but this is only the distance between the clusters and not the actual location of sensor nodes, considering the random selection of member sensors inside the cluster. Therefore, our relocation protocol considering the distance between the sensor nodes has obtained various research results which cannot be compared with the previous researches.

**Figure 12:**
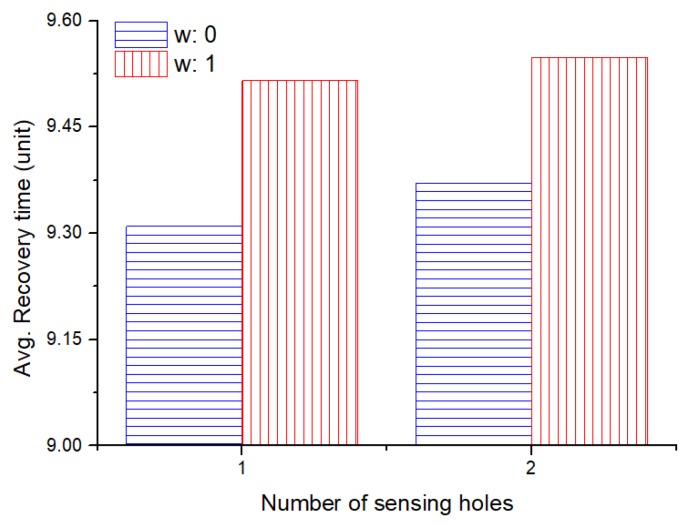
The average recovery time according to weight *w*.

As shown in Figure 12, it can be seen that the simulation for recovering the sensing hole is very fast, considering the rearrangement of the hopping sensors with a weight close to zero (i.e., distance only). In contrast, simulation results with a weight close to 1 that only takes into account the hopping capability will be the slowest. However, in a rearrangement of the hopping sensor nodes, it cannot be determined whether considering only the distance is desirable in order to reduce the recovery time. In fact, it is also important to consider the energy for movement, since sensor nodes are inherently energy-limited. Therefore, various simulation results below are mandatory to see how these weights affect the results.

**Figure 13:**
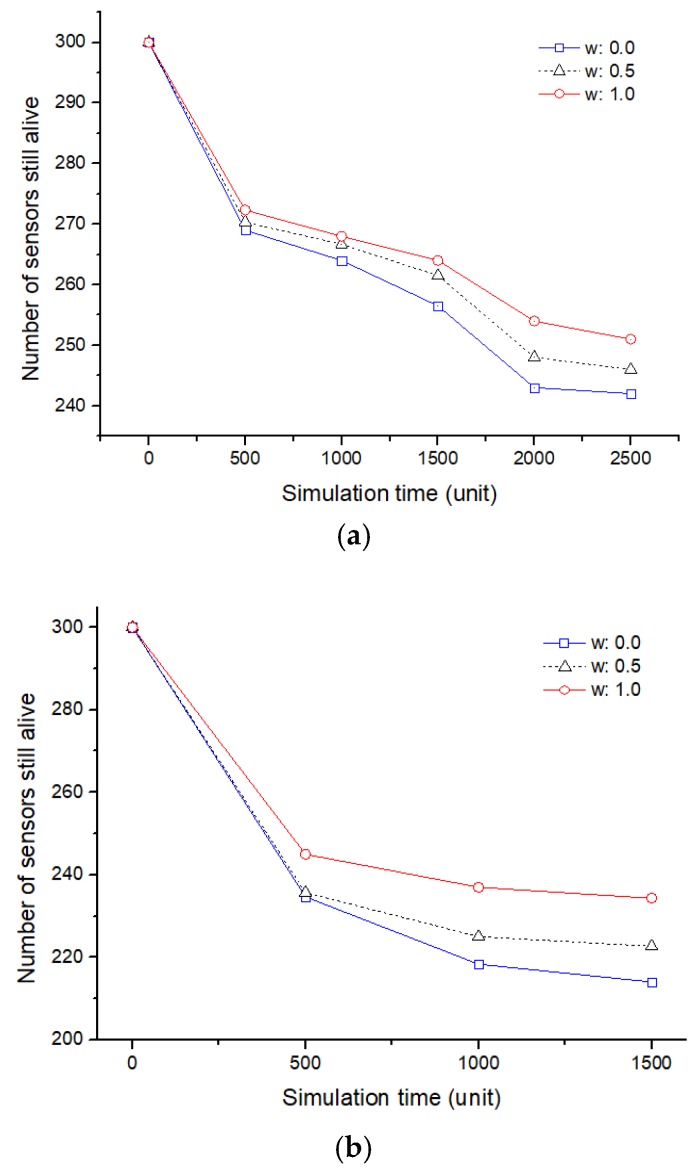
The number of total hopping sensors still alive: (**a**) A single sensing hole occurred; (**b**) two sensing holes occurred.

As shown in Figure 11, sensor nodes in a specific cluster continuously collecting data lead to a failure (yellow marked). In order to recover such a sensing hole, a scenario in which the hopping sensors move is repeated over time. Figure 13 shows the number of movable sensors over the entire network according to the simulation time. A fault in a hopping sensor node is not simply determined by the depletion of energy due to collecting data. If the sensor is impossible to move due to frequent movement, it is also regarded as a fault of the hopping sensor node. The number of surviving nodes is the largest when only mobility (energy for movement) is considered. However, we cannot conclude that considering the possibility of movement is the best for sensing hole recovery. The following results supplement this explanation.

**Figure 14:**
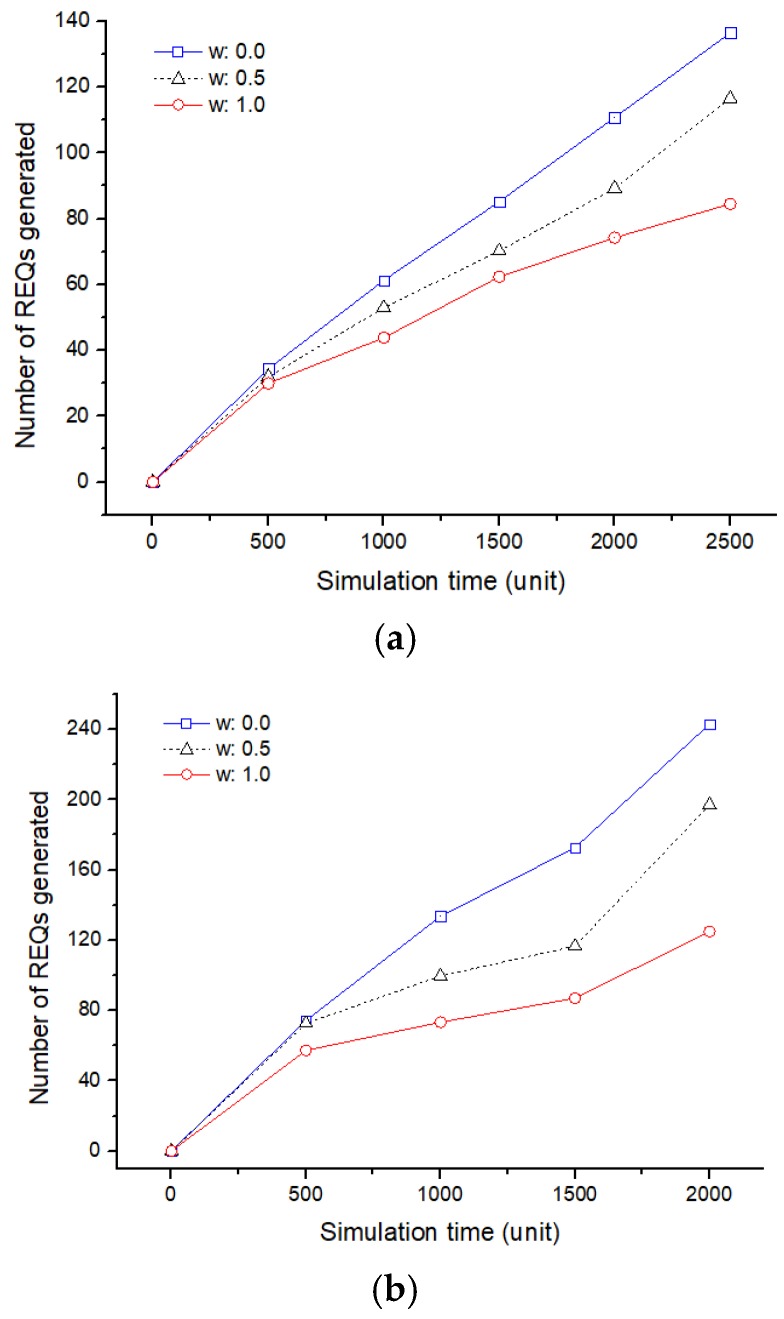
The number of total REQ messages generated: (**a**) A single sensing hole occurred; (**b**) two sensing holes occurred.

In Figure 14, the number of REQs, which is a hopping sensor movement request message for a sensing hole recovery, is analyzed according to the simulation time. Briefly, considering the energy, the frequency of occurrence of REQ is decreased. In contrast, the frequency of REQ is increased considering distance only. The reason for this is as follows:

First, the occurrence of REQ generally means that a sensing hole is detected. If a node is defective due to continuous data collection in a specific cluster zone, it is possible to speed up the recovery time considering only the distance. However, since the sensing hole appears again after the fast recovery, the number of REQs should be increased.

Second, from a different point of view, there is a possibility that defects may occur as the sensor moves, since only the distance is taken into consideration and the energy is not considered. That is, a new REQ may be generated again if the number of available sensors is less than the number of sensors requested by the previous REQ.

It is also worthy to note that the definition of the sensing hole means that there are less than a predetermined number of sensors in the cluster zone and does not mean that there is no sensor for data collection. Therefore, it cannot be concluded that the fast recovery of a sensing hole is the most important, considering only the position. That is, selecting a sensor that can survive longer even if the recovery time is little slower and rearranging it will be one of the new approaches.

**Figure 15:**
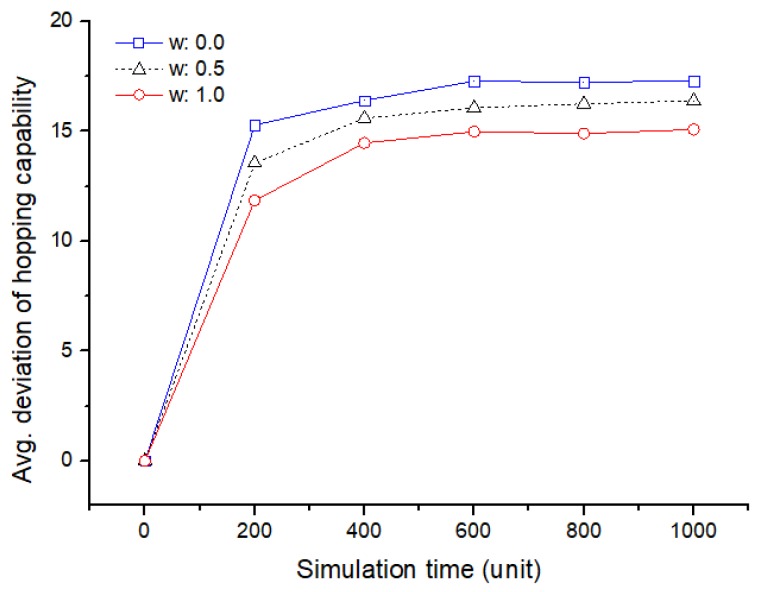
The average variance of hopping capability for a single sensing hole.

Because of the limited energy of the wireless sensor node, relocation without consideration of the energy may generate more sensing holes and lead to network disconnection. Thus, it is very important to consider the saving of the moving energy of the hopping sensor every rearrangement. Figure 15 shows the average deviation of the residual energy among the deployed member sensor nodes with the experience of movement, according to the simulation time. The relocation of the member sensor node considering only the shortest distance confirms that the residual deviation of the mobile energy becomes larger as seen in Figure 15. As mentioned above, it can be interpreted that if the residual deviation increases, the ability to recover the sensing hole decreases. Therefore, it can be concluded that a relocation technique considering distance and energy is very important according to importance of recovery time.

## 4. Conclusions

Sensors are randomly distributed and the desired data is collected by the sensors in order to characterize areas that are difficult to access. However, due to the energy limitations of the sensor nodes, sensing holes could be generated, and then a network disconnection is also caused. In order to recover the sensing hole, the rearrangement of the hopping sensor node is considered in this paper. Most of previous relocation protocols have been based on the shortest path between a cluster header which can move hopping sensors and a sensing hole header. However, it is practically impossible that the cluster header of the sensing hole already knows the location of all the cluster headers of a given network.

In this paper, we overcome the limit of requirements of the network topology information and propose new relocation protocol in a distributed environment which is very suitable for actual cases. Considering the characteristics of the collected data, designing the relocation protocol of the sensors to be able to consider both the distance to the sensing hole and the energy which enables the hopping sensors to move is the major achievement of the proposed methods. As far as we know, this study is the first simulation using OMNeT++ which reflects actual environments. Future research on the relocation protocol considering various geographical environments will further improve the proposed protocol.

## Figures and Tables

**Table 1:** The simulation environment.

Network Area	250 m × 150 m
Number of total member sensor nodes scattered	285
Number of cluster headers	15
Number of minimum members in each cluster zone	5
Maximum communication radius by jumping	29 m
Maximum distance moved by one hopping	1 m
Hopping capability per a sensor initially	290
